# Is Calcium Score in the Abdominal Aorta or Renal Arteries Predictive of Acute Kidney Injury After Cardiopulmonary Bypass: An Exploratory Study

**DOI:** 10.7759/cureus.31466

**Published:** 2022-11-13

**Authors:** Ludmil V Mitrev, Pauline Germaine, Connor Crudeli, Anthony Santisi, Aditi Trivedi, Noud Van Helmond, John Gaughan

**Affiliations:** 1 Department of Anesthesiology, Cooper University HealthCare, Camden, USA; 2 Anesthesiology, Cooper Medical School of Rowan University, Camden, USA; 3 Department of Radiology, Cooper University HealthCare, Camden, USA; 4 Radiology, Cooper Medical School of Rowan University, Camden, USA; 5 Research, Cooper Medical School of Rowan University, Camden, USA; 6 Cooper Research Institute, Cooper University HealthCare, Camden, USA

**Keywords:** calcium score, cardiopulmonary bypass, hypertension, pulse pressure, acute kidney injury

## Abstract

Introduction

Acute kidney injury (AKI) remains a serious complication after surgery with cardiopulmonary bypass (CPB). A relationship similar to the one between coronary artery calcification and increased incidence of cardiac complications is hypothesized to exist for aortic calcification and the development of AKI. Elevated pulse pressure (PP) hypertension has been shown to be a predictor of AKI-CPB (AKI after CPB surgery), and calcium deposition and stiffening of the body’s conduit arteries may be part of this process. We hypothesized that calcium scores obtained from non-contrast computed tomography (CT) scans of the infrarenal aorta and renal arteries would be independently and significantly associated with AKI-CPB.

Methods

We conducted a retrospective study of 65 subjects who underwent non-emergent open heart surgery with CPB in a tertiary healthcare center. AKI-CPB was diagnosed using the Acute Kidney Injury Network criteria. Aortic and renal artery calcium (Agatston) scores were obtained and entered into a multivariable logistic regression model alongside other significant predictors of AKI-CPB from a univariable analysis.

Results

Pulse pressure, body surface area, and pre-operative serum creatinine were significantly associated with the development of AKI-CPB, but the calcium scores were not. For PP, the odds ratio (OR) was 1.062, (95% Wald confidence interval {CI}=1.012 - 1.114). The OR for the calcium score in the aorta was 1.0000 (95% CI=1.0 - 1.0).

Conclusions

Agatston calcium scores in the renal arteries and infrarenal aorta were not independently associated with AKI-CPB, but arterial stiffening, as indicated by elevated pulse pressure, was predictive of AKI-CPB.

## Introduction

Acute kidney injury remains a serious complication after surgery with cardiopulmonary bypass (AKI-CPB). By some estimates, it occurs in 20 to 70% of cases, with the exact incidence depending in part on the definition of AKI [1,2]. It is associated with increased morbidity, mortality, and healthcare costs [3,4]. Numerous predictors of AKI-CPB have been described, many of which are non-modifiable. One such predictor is elevated pulse pressure (PP) hypertension, which can be seen as a marker of arterial senescence and stiffening [5-8]. A positive correlation exists between calcification in the renal arteries, hypertension, and renal disease [9-11]. Oxygen delivery index (DO_2_I) has also been shown to be independently associated with AKI in retrospective studies and in one prospective, randomized trial [12-16]. In this exploratory study, we hypothesized that calcium scores obtained from non-contrast-enhanced computed tomography scans of the aorta and renal arteries would be independently and significantly associated with AKI when analyzed alongside other predictors of AKI-CPB in a logistic regression model.

## Materials and methods

Between 2015 and 2019, our group developed a retrospective, observational dataset of patients undergoing coronary artery bypass grafting (CABG), valve, or CABG + valve open heart surgery with CPB in a tertiary healthcare center in the United States. Post-operative AKI-CPB was scored using the Acute Kidney Injury Network (AKIN) criteria [17]. CPB was performed using centrifugal arterial pumps, low-circuit priming volumes, and ultra-low prime oxygenators. DO_2_I was calculated retrospectively based on arterial blood gas sample results, including arterial hemoglobin values and oxygen saturation, performed no less than every 30 minutes during CPB. DO_2_I was derived for each 30-minute epoch between blood gases by using the formula for oxygen delivery: (1.34*Hgb*SaO_2_+0.003*PaO_2_)*pump flow, divided by body surface area (BSA) and multiplied by a factor of 10 to convert the unit of measurement to L/min/m^2^ (Hgb, hemoglobin in grams/dL; SaO_2_, arterial oxygen saturation; PaO_2_, arterial oxygen tension in mmHg; pump flow in L/min; and BSA in m^2^). We used the Mosteller formula to calculate BSA [18].

After obtaining approval from our Institutional Review Board (approval number 19-307), the medical records of the subjects in this dataset were examined for the presence of non-contrast abdominal computed tomography (CT) scans performed for any reason within the 12-month period preceding or following the subjects’ index cardiac surgery. Using CT scans performed post-surgery was considered acceptable, as calcium deposition and arterial aging is a slow process that, if significant, likely had begun well ahead of the surgery. This enabled the development of a larger sample. All retrieved scans were performed on a Toshiba Aquillion 64 Slice CT scanner (Canon Medical Systems USA Inc., Tustin, CA) The presence and extent of calcifications in the infrarenal abdominal aorta and the renal arteries were assessed utilizing an independent Aquarius workstation (TeraRecon, Durham, NC) and an Agatston score was calculated [19]. Calcifications were defined as regions of high density >130 Hounsfield Units (HU) and as areas of 3 or more contiguous pixels. The length of each renal artery was evaluated from the ostium to the renal hilum for the presence of calcification. The infrarenal abdominal aorta was evaluated from the renal ostium to the bifurcation.

Statistical analysis

Factors known to be predictive for AKI-CPB (dichotomous) were evaluated separately for their relationship with AKI-CPB using logistic regression. Pre-operative variables included age, history of congestive heart failure (CHF), history of myocardial infarction (MI), diabetes mellitus, beta-blocker (BB) use, serum creatinine, angiotensin-converting enzyme inhibitor (ACE-I) or angiotensin receptor blocker (ARB) use, BSA, and PP. Intra-operative independent variables in the univariable analysis were duration of CPB, inotrope use (epinephrine, dobutamine, or milrinone), and mean DO_2_I. Calcium scores in the right and left renal arteries (CS-RRA and CS-LRA) and infrarenal aorta (CS-Aorta) were obtained as described above. 

Odds ratios (OR), 95% confidence intervals (CI), and p-values were calculated to estimate the quantitative risk for the development of AKI-CPB. Only those parameters found to be significantly associated with AKI-CPB in the univariate analysis (p<0.05) were entered into a multivariable logistic regression model. A p-value of 0.05 was used (two-sided).

## Results

**Figure 1:**
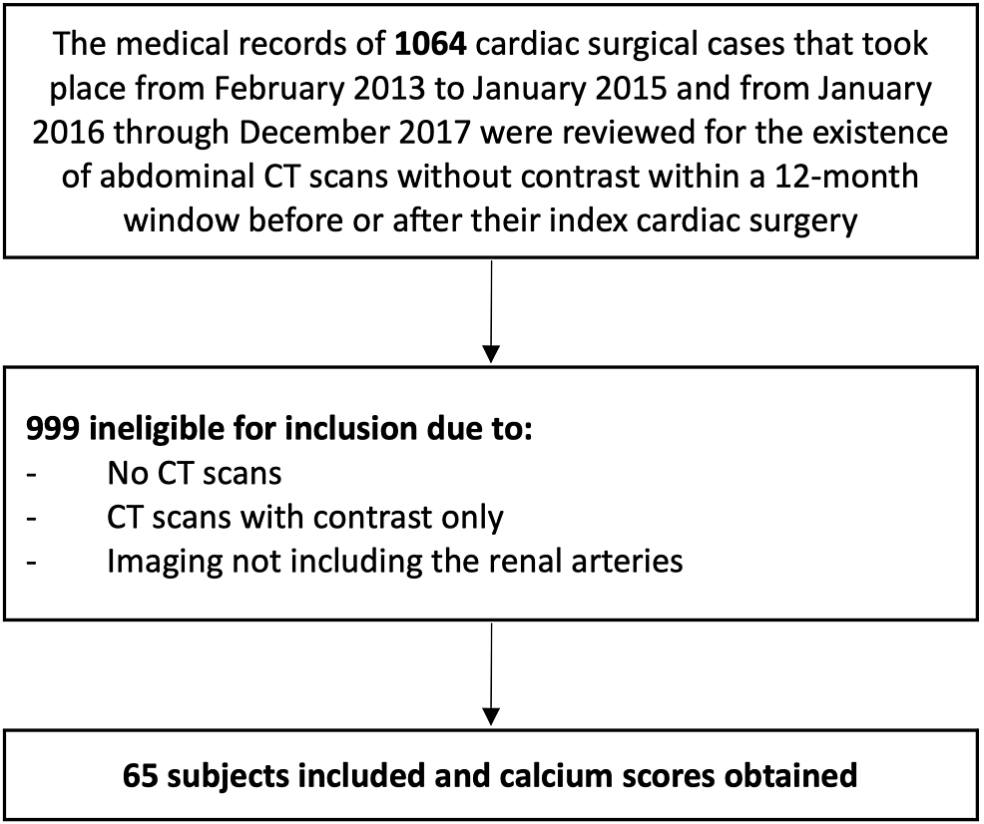
Sample Development Flowchart

Figure 1 shows a subject inclusion flowchart. Table 1 shows the baseline characteristics of the subjects in the final sample.

**Table 1:** Preoperative Characteristics of the Study Subjects BMI: body mass index (Mosteller formula); CKD: chronic kidney disease; MI: myocardial infarction; COPD: chronic obstructive pulmonary disease; CABG: coronary artery bypass graft; ACE: angiotensin-converting enzyme; ARB: angiotensin receptor blocker

Preoperative Characteristics	Frequency	Percent of population
Age		
≤50	7	11
51-60	11	17
61-70	22	34
71-80	17	26
>80	8	12
Gender		
Male	34	52
Female	31	48
BSA		
<1.50	3	5
1.50-2.00	37	57
>2.00	25	38
Medical History		
Prior MI	16	25
Diabetes mellitus	29	45
Hypertension	56	86
Smoking	43	66
Peripheral vascular disease	16	25
Congestive Heart Failure	29	45
Asthma/COPD	20	31
Previous CABG	1	2
Previous valve surgery	3	5
Aortic atherosclerosis	24	37
Left ventricular hypertrophy	23	35
Atrial fibrillation	5	8
Preoperative Medications		
Beta blocker	35	54
ACE/ARB	17	26
Calcium channel blocker	10	15
Diuretics	19	29
Direct vasodilators	7	11
Preoperative Creatinine		
≤0.75	19	29
0.76-1	17	26
1.01-1.25	14	22
1.26-1.50	7	11
>1.51	7	11
Missing	1	2
Preoperative Pulse Pressure		
≤40	5	8
41-60	22	34
61-80	22	34
81-100	11	17
>100	5	8

Twenty-seven patients (41.5%) in the sample developed AKI-CPB stages 1-3 (Table 2). The mean DO_2_I was 253 mL/min/m^2^ (range, 115 - 420 mL/min/m^2^). In univariable analysis, pre-operative creatinine and BB use, BSA, PP, CS-Aorta, and DO_2_I were significantly associated with the development of AKI-CPB. The odds ratio (OR) for CS-Aorta was 1.136 (95% confidence interval (CI): 1.022 - 1.262, p=0.0182). Age, history of congestive heart failure (CHF), diabetes mellitus, history of MI, pre-operative use of ACE-I or ARB, duration of CPB, and intraoperative inotropic agents were found not to be significantly associated with AKI-CPB. CS-LRA and CS-RRA were also not significantly associated with AKI-CPB. Table 3 summarizes the results of the univariable analysis.

**Table 2:** Post-Operative Acute Kidney Injury Outcomes in the Study Sample

Acute Kidney Injury Stage	Frequency	Percent
0	38	58.5
1	10	15
2	5	8
3	12	18.5

**Table 3:** Results of the Univariable Analysis. CHF: congestive heart failure; MI: myocardial infarction; DM: diabetes mellitus; BB: beta blocker; ACE: angiotensin converting enzyme; ARB: angiotensin receptor blocker; BSA: body surface area; PP: pulse pressure; CPB: cardiopulmonary bypass; CS-RRA: calcium score in the right renal artery; CS-LRA: calcium score in the left renal artery; CS-Aorta: calcium score in the abdominal aorta; DO_2_I: mean oxygen delivery index during CPB

Parameter	Odds Ratio	95% Wald Confidence Limits	p-value
Age	1.038	0.994 - 1.084	0.0922
History of CHF	1.277	0.473 - 3.444	0.6293
History of MI	3.137	0.973 - 10.114	0.0556
DM	1.277	0.473 - 3.444	0.6293
Pre-operative BB use	3.266	1.146 - 9.308	0.0268
Pre-operative serum creatinine	12.016	2.128 - 67.840	0.0049
Pre-operative ACE/ARB use	1.357	0.445 - 4.135	0.5915
BSA	32.343	3.059 - 341.961	0.0039
Pre-operative PP	1.030	1.004 - 1.058	0.0244
Duration of CPB	1.006	0.994 - 1.019	0.3467
Intra-operative inotrope use	1.058	0.388 - 2.881	0.9125
CS-RRA	1.280	0.959 - 1.707	0.0935
CS-LRA	1.004	0.999 - 1.008	0.0829
CS-Aorta	1.136	1.022 - 1.262	0.0182
DO_2_I per 100 mmHg	0.296	0.092 – 0.947	0.0402

The variables that were significantly associated with AKI-CPB were then entered into a multivariable model (Table 4). PP remained significantly associated with the development of AKI-CPB, but DO_2_I and the calcium scores did not (p > 0.05). For PP, the OR=1.062, (95% CI=1.012 - 1.114, p=0.0144). This would seem to indicate that for each mmHg rise in baseline PP, there was a 6.2% increase in the odds of the patient developing AKI-CPB. The other variables that were significantly predictive in the multivariable model were BSA and pre-operative creatinine. 

**Table 4:** Results of the Multivariable Analysis BB: beta blocker; BSA: body surface area; PP: pulse pressure; CS-Aorta: calcium score in the abdominal aorta; DO_2_I: oxygen delivery index during CPB

Parameter	Odds Ratio	95% Wald Confidence Limits	p-value
Pre-operative BB use	2.189	0.431 - 11.110	0.3447
BSA per 0.1 m^2^	2.063	1.308 - 3.255	0.0018
Pre-operative PP	1.062	1.012 - 1.114	0.0144
Pre-operative serum creatinine per 0.1 mg/dL	1.503	1.110 – 2.035	0.0084
CS-Aorta	1.000	1.000 - 1.000	0.2288
DO_2_I	0.989	0.972 – 1.006	0.2166

## Discussion

Our retrospective cohort study in cardiac surgical patients undergoing CABG, valve, or combined surgery with CPB found that calcium scores in the renal arteries or infrarenal aorta were not independently and significantly associated with the development of AKI-CPB, but PP, pre-operative creatinine, and BSA were associated with this outcome. This ran contrary to our initial hypothesis. 

Arterial calcification has been shown to be more common in patients with CKD as demonstrated by the 1996 milestone paper by Braun et al. [20]. Subsequent studies have suggested an independent correlation between vascular calcification and renal function. A relationship similar to the one between coronary artery calcification and increased incidence of cardiac complications is hypothesized to exist for aortic calcification and the development of AKI [21]. The suspected mechanism involves arterial stiffening as a consequence of calcium deposition in the aorta, reducing the elastic compensatory reflex during times of hypoperfusion, essentially the same mechanism as the one associated with the existence of elevated PP [21]. This premise provided our rationale to study the aortic calcium score to determine if it might be an independent risk factor for AKI-CPB.

There could be several reasons why this study did not find an association between the calcium score and AKI-CPB. Our finding could be related to the small sample size and the fact that our outcome variable was AKI in the immediate post-operative period (five to seven days). The incidence of AKI or chronic renal disease later in life for the subjects in our sample is not known. Furthermore, we used the “raw” Agatston scores as predictors in this study, as opposed to a score of calcific aortic disease burden such as the one recently described by Reddy et al. [22]. This group derived a total score of abdominal aortic calcification (AAC) from sagittal CT reformats of the aorta distal to the inferior mesenteric artery (IMA) and showed it to have a high interrater agreement (interclass correlation), as well as a strong correlation with the corresponding CT calcium score. It would be interesting to examine whether this score is associated with AKI-CPB.

We used the calcium score in the infrarenal aorta because calcific deposits in the lower aorta occur earlier in life [23,24]. Furthermore, when calcific deposits are present in higher segments of the abdominal aorta, they are accompanied by extensive calcifications in the lower part of the aorta.

Our study appears to confirm the findings of other authors that PP is significantly and independently associated with the development of AKI-CPB. PP is a somewhat overlooked but well-substantiated independent risk factor for adverse renal outcomes such as AKI after surgery with CPB [5-7,25]. Elevated pulse pressure is reflective of the loss of arterial elastance in conduit arteries, and may be contributory to end-organ dysfunction (AKI, left ventricular hypertrophy, atherosclerosis, MI, CHF) [5,26-29]. DO_2_I did not reach statistical significance as a predictor of AKI-CPB, possibly due to the limitations of the sample size. However, other authors have shown it to be associated with AKI-CPB. Ranucci et al. demonstrated in a randomized clinical trial that maintaining DO_2_I at or above 280 mL/min/m^2^ reduced AKI-CPB stage 1, but not stage 2 and 3, when defined according to the AKIN criteria [16]. Newland et al. were able to demonstrate in a sample of 19,410 CPB cases that a 10 mL/min/m^2^ reduction in DO_2_I was associated with an average increase in the odds of AKI-CPB of 7% [15]. The DO_2_I threshold that showed optimal diagnostic accuracy for AKI-CPB in their sample was 270 mL/min/m^2^. 

Our study was not spared the shortcomings of small, retrospective studies. The sample size was limited by the availability of CT-scan data. Our cohort excluded emergency cardiac surgery and the use of an intra-aortic balloon pump. We did not evaluate the influence of pre-operative or intra-operative nadir hemoglobin, but assumed its effect on the odds of developing AKI-CPB to be mediated via DO_2_I. The Ranucci et al. trial also found that erythrocyte transfusion in the ICU was independently associated with stage 1 AKI-CPB. Our sample did not include data on post-operative transfusion, and this was not evaluated. We did not possess data on pre-operative contrast load or inflammatory markers such as high-sensitivity C-reactive protein, and those were not taken into account in the analysis.

## Conclusions

In a single-center retrospective study of cardiac surgical patients undergoing CABG, valve or combined surgery with CPB, PP, pre-operative creatinine, and BSA were significantly and independently associated with the development of AKI-CPB, while Agatston calcium scores in the renal arteries or infrarenal aorta were not.
